# Isoflavonoids and Epigenetic Modulation: Therapeutic Insights for Cancer Treatment

**DOI:** 10.1002/cbdv.202503446

**Published:** 2026-02-16

**Authors:** Eduardo de Moraes e Sousa, Maria Claudia dos Santos Luciano, Gabriel Caetano de Souza, Maria Francilene Souza Silva, Fátima de Cássia Evangelista de Oliveira, Sarah Sant'Anna Maranhão, Felipe Vasconcelos, Cristiana Libardi Miranda Furtado, Claudia do Ó Pessoa

**Affiliations:** ^1^ Northeast Biotechnology Network—RENORBIO Recife Brazil; ^2^ Drug Research and Development Center, Department of Physiology and Pharmacology Federal University of Ceará Fortaleza Brazil; ^3^ Ceará Cancer Institute—ICC/Rodolfo Teófilo Faculty—F Fortaleza Brazil; ^4^ Department of Translational Medicine Federal University of Ceará Fortaleza Brazil; ^5^ Núcleo De Biologia Experimental (Nubex) Fortaleza Brazil; ^6^ Department of Genetics Ecology and Evolution Institute of Biological Sciences Federal University of Minas Gerais Belo Horizonte Brazil

**Keywords:** epidrugs, epigenome, flavonoids, genetics, natural products

## Abstract

Isolavonoides represent the second largest subgroup of flavonoids and have an influence on critical molecular pathways and restore cellular homeostasis, through the reprogramming of epigenetic regulatory mechanisms. This feature indicates a crucial therapeutic potential that could be better explored to attend cancer treatment. Isoflavonoids, acting as epigenetic modulators, could contribute to the development of new therapeutic approaches in cancer, especially in onco‐hematological diseases. Pterocarpans are a subgroup of isoflavonoids that have been extensively studied for their biological properties. The molecule (+)‐2,3,9‐trimethoxypterocarpan demonstrates high gastrointestinal (GI) absorption and the ability to cross the blood–brain barrier (BBB) in silico without violating Lipinski's rule, making it a desirable candidate in leukemia treatment. The synthesis of this molecule dates back more than a decade. In silico models, such as SwissADME, corroborate the notion of good intestinal absorption and the ability to cross the BBB. Also, it is suggested that P‐glycoprotein is a substrate, which is related to its potential for active efflux from both the BBB and GI. This review highlights the biological mechanisms of this class of natural products from a translational perspective, emphasizing their chemical properties and epigenetic biological activities, which offer new therapeutic perspectives, particularly in oncology.

## Graphical abstract

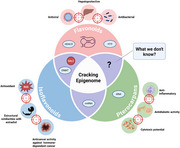

Venn diagram illustrating the epigenetic roles of three phytochemical groups—flavonoids, isoflavonoids, and pterocarpans.

## 1 Introduction

The flavonoids are related to diverse roles, specifically targeting the epigenome of tumor cells in human cancers [1], including effects on important epigenetic modifications such as histone deacetylation [2, 3] and DNA methylation [3]. Computational (in silico) evaluations consider multiple parameters that help to optimize the screening of potential therapeutic agents, especially considering the absorption, distribution, metabolism, excretion, and toxicity (ADMET) properties, key determinants of pharmacological efficacy and safety [4].

Leukemias are a group of hematological malignancies of the blood in which the clonal expansion of aberrant hematopoietic stem cells causes malignant disorders of the blood and bone marrow [5]. Disruptions in chromatin modifiers and remodelers have been implicated in hematological malignancies, corroborating and strengthening the pivotal role of epigenetic aberrations in the etiology of cancer. Compounds targeting epigenetic enzymes (epidrugs) are the next‐generation models of drugs that have been considered in our understanding and use in different cancer epigenetics approaches [6, 7].

The application of predictive software in the early stages of drug discovery enables in silico analysis of new molecules. This strategy has several advantages, including low cost and reliability in obtaining ADME data. These predictions make the process more efficient by supporting informed decisions on which compounds should advance through the development stages. For this reason, we chose to evaluate the pharmacokinetic properties of the molecules described in this review study.

This review highlights the potential of isoflavonoids (IFs) in modulating epigenetic mechanisms that regulate gene expression. Flavonoids are a well‐known class of compounds with several biological activities; however, the epigenetic potential of certain flavonoid‐derived subclasses, such as IFs, is an important area of discussion and remains largely unexplored.

### 1.1 Targeting Epigenetics in Cancer

Epigenetic regulation refers to a reversible mechanism that modulates gene expression and chromatin structure without altering the underlying DNA sequence. This process primarily involves several covalent modifications to nucleic acids and histone proteins [8]. The main epigenetic regulatory mechanisms include DNA methylation, histone modification—such as acetylation, methylation, ADP‐ribosylation, ubiquitination, and phosphorylation—and the regulation mediated by noncoding RNA (ncRNA) [1, 9, 10].

Environmental factors are well‐recognized epigenetic modifiers that influence epigenetic reprogramming and play an essential role in both the establishment and maintenance of epigenetic markers [11].

Given their critical roles in modulating gene expression and maintaining chromosomal stability, disruptions of epigenetic mechanisms have significant consequences for numerous physiological and pathological processes. Such disturbances are associated with epigenetic disorders, including neurodevelopmental syndromes and cognitive impairments [12]. Epigenetic alterations are more flexible than genetic mutations. This enables them to play crucial roles in immune surveillance and drug resistance development [12].

Epigenetic reprogramming is a critical event in the process of cell differentiation. Once established, epigenetic patterns of genomic function must be maintained during cell division and DNA replication at the expense of cell fate. Disruption of the epigenetic landscape in differentiated cells can lead to altered cell fate that promotes the development of carcinogenesis and enhances tumor progression [13].

The identification of the cell of origin of leukemia has remained a longstanding challenge in cancer research over the last few decades. Oncogene‐induced reprogramming and the mixed lineage leukemia gene (MLL), including chimeric fusion, partial tandem duplication (PTD), amplification, and internal exonic deletion, represent one of the most common recurring oncogenic events [22, 23].

Given the possibility of reprogramming the epigenome and using it to alter the cellular landscape, it could prove to be a promising and up‐to‐date therapeutic strategy [7], especially in leukemia treatment. Epigenetic drugs, or simply epidrugs, are chemical compounds that cause changes in DNA and chromatin structure. They act on the enzymes responsible for the maintenance and establishment of epigenetic modifications, primarily by inhibiting DNA methyltransferases (DNMTs) and histone deacetylases (HDACs), resulting in the reactivation of epigenetically silenced genes involved in tumor suppression and DNA repair [16, 17].

Although the development, research, and application of epidrugs is extensive, they have a compelling antitumor effect that underscores their utility in cancer treatment. The development and research of biomarkers for epidrugs offer a promising direction for screening new antineoplastic agents.

### 1.2 Flavonoids: Key Molecular Features and Signaling Pathways

Flavonoids are plant‐derived secondary metabolites commonly found in the Fabaceae Lindl. (Leguminosae) family [18]. They are synthesized in response to environmental stressors, including pathogen attack, insect herbivory, extreme temperature fluctuations, droughts, salinity, floods, and heavy metals. These metabolites play a vital role in plant protection and are distributed throughout various plant tissues, including fruits, grains, bark, and stems [19, 20, 21].

**FIGURE 1:**
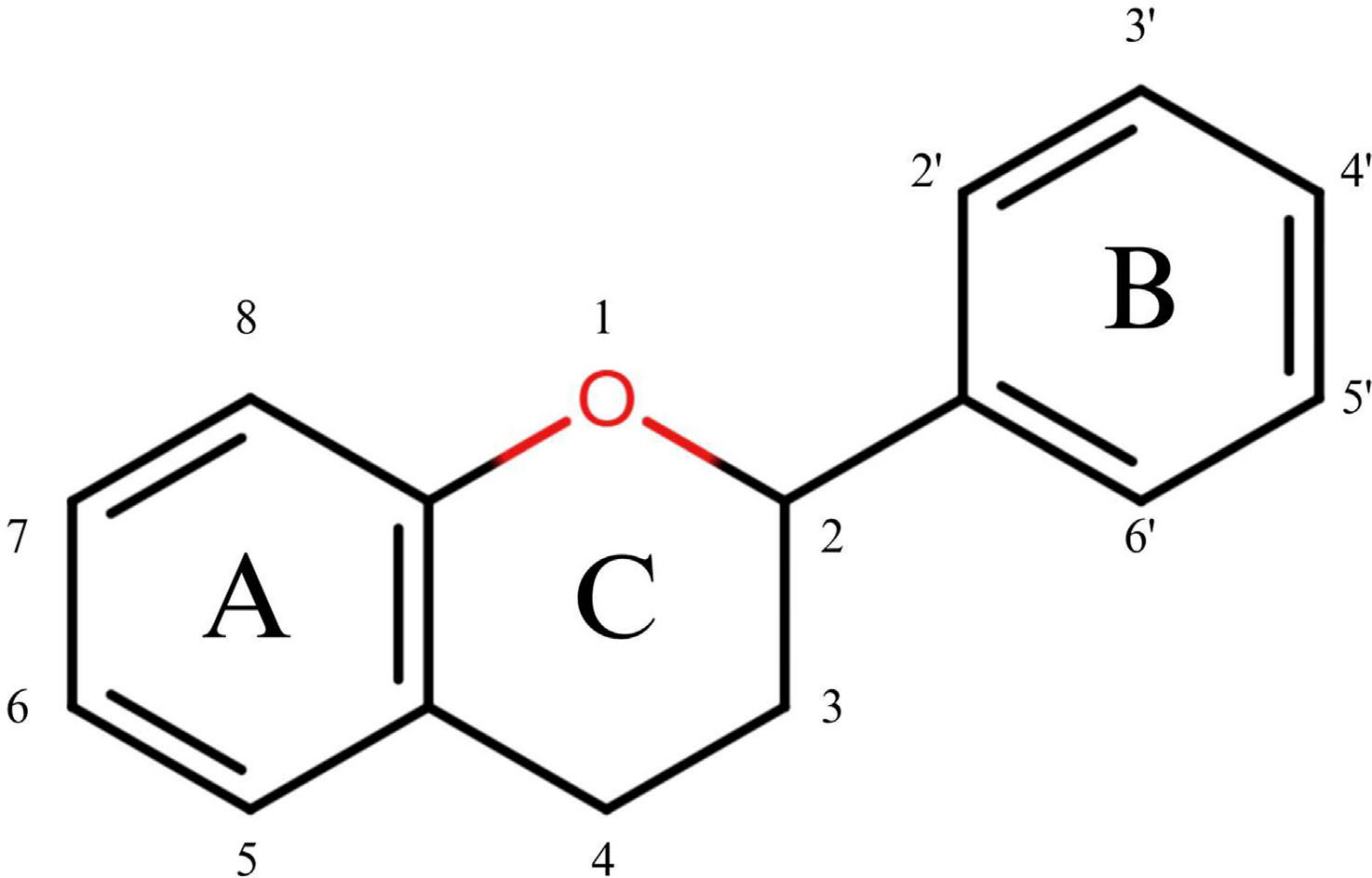
Basic flavonoid structure: 15‐carbon skeleton consisting of two benzene rings (A and B) linked via a heterocyclic pyrane ring (C), forming a three‐carbon bridge (C6–C3–C6).

Among polyphenolic compounds, flavonoids represent one of the most prominent subclasses, notable for their structural diversity and secondary metabolites, with a wide range of biological activities [22]. The basic structure of flavonoids is based on a C6–C3–C6 skeleton typically occurring as an aglycone with a benzo‐γ‐pyrone core (Figure 1). Based on the position of the phenyl group on the central pyrone ring, flavonoids can be classified into two major groups: flavonoids at the 2‐position and IFs at the 3‐position.

Flavonoids are associated with a variety of health‐promoting benefits and are used as ingredients in various pharmaceutical, nutraceutical, cosmetic, and medicinal products. In addition, flavonoids exhibit a broad spectrum of pharmacological activities: antiviral, hepatoprotective, antibacterial, analgesic, cytostatic, anti‐allergic, anti‐estrogenic, estrogenic, and apoptotic properties [19, 23, 24]. Since 2010, flavonoids have been repeatedly recognized as important chemopreventive phytochemicals for age‐related diseases [25, 26, 27].

Numerous natural compounds isolated from medicinal plants, such as flavonoids, phenolic acids, stilbenes, and ketones, have demonstrated the ability to prevent and treat a wide range of human diseases, including cancer. These effects are, in part, mediated through their action on epigenetic modifiers, such as activation or suppression of the expression of specific genes at different levels. Histone methyltransferases (HMT), DNMTs, histone acetyltransferases (HATs), and demethylases modify and maintain epigenetic signals that influence chromatin structure and cellular functions. Dysregulation of these proteins is linked to various diseases, particularly malignant tumors [28, 29].

Several reports suggest that flavonoids have the potential to restore epigenetic marks that become dysregulated during carcinogenesis [30, 31, 32]. Among them, epigallocatechin gallate (EGCG), a flavonoid found in green tea, stands out for its antiproliferative activity against various cancer cell lines, such as HCT‐116, HT29, MCF‐7, and MDA‐MB‐231, while it did not show antiproliferative activity against nontumorigenic epithelial MCF10A cells. In all the studies mentioned, EGCG treatment has contributed directly and indirectly to epigenetic regulation [2]. Ganai et al. [32] have reviewed scientific reports from the 1990s until 2020 on the antiproliferative effects of luteolin and its potential use in epigenetic‐based therapies. Zhu et al. [33] have emphasized the potential of quercetin for epigenetic regulation, including effects on several DNMTs, inhibition of DNMT1, DNMT3A, and DNMT3B, and enhancement of histone acetylation, especially on histones H3 and H4. To date, it is estimated that there are approximately 5000 known flavonoid structures, which can be categorized into several groups based on their chemical structure, degree of unsaturation, and oxidation state of the C‐ring, including flavanones, flavones, flavonols, chalcones, flavanols, anthocyanidins, and IFs [18, 19, 24, 34]_._

Studies indicate that flavonoids exert anticancer effects by modulating apoptotic, cell cycle, and signaling pathways. Daidzein is a flavonoid that has been experimentally shown to reduce ANXA1 levels and downregulate COX‐2 activity, leading to cell cycle arrest, induction of apoptosis, and enhanced phagocytosis [35]. Similarly, flavonoid‐rich bergamot juice extract acts on the SIRT2/AKT/TP53 axis by reducing AKT phosphorylation, SIRT2 downregulation, TP53 upregulation, and affecting Wnt5a and β‐catenin signaling, culminating in S‐phase cell cycle arrest and apoptosis [36]. Luteolin has also been widely reported to induce apoptosis via the mitochondrial pathway, characterized by BAX upregulation, BCL‐2 downregulation, DNA fragmentation, and morphological differentiation toward granulocytes (Table S1) [32].

Collectively, these findings highlight flavonoids as bioactive compounds that act in the central signaling pathways such as β‐catenin and PI3K–Akt [35, 36]. The Wnt/β‐catenin pathway embraces a group of proteins that are directly related to the regulation of adult tissue homeostasis [37], while AKT plays a significant role in various cellular processes, including metabolism, cell proliferation, survival, and growth [38, 39].

**FIGURE 2:**
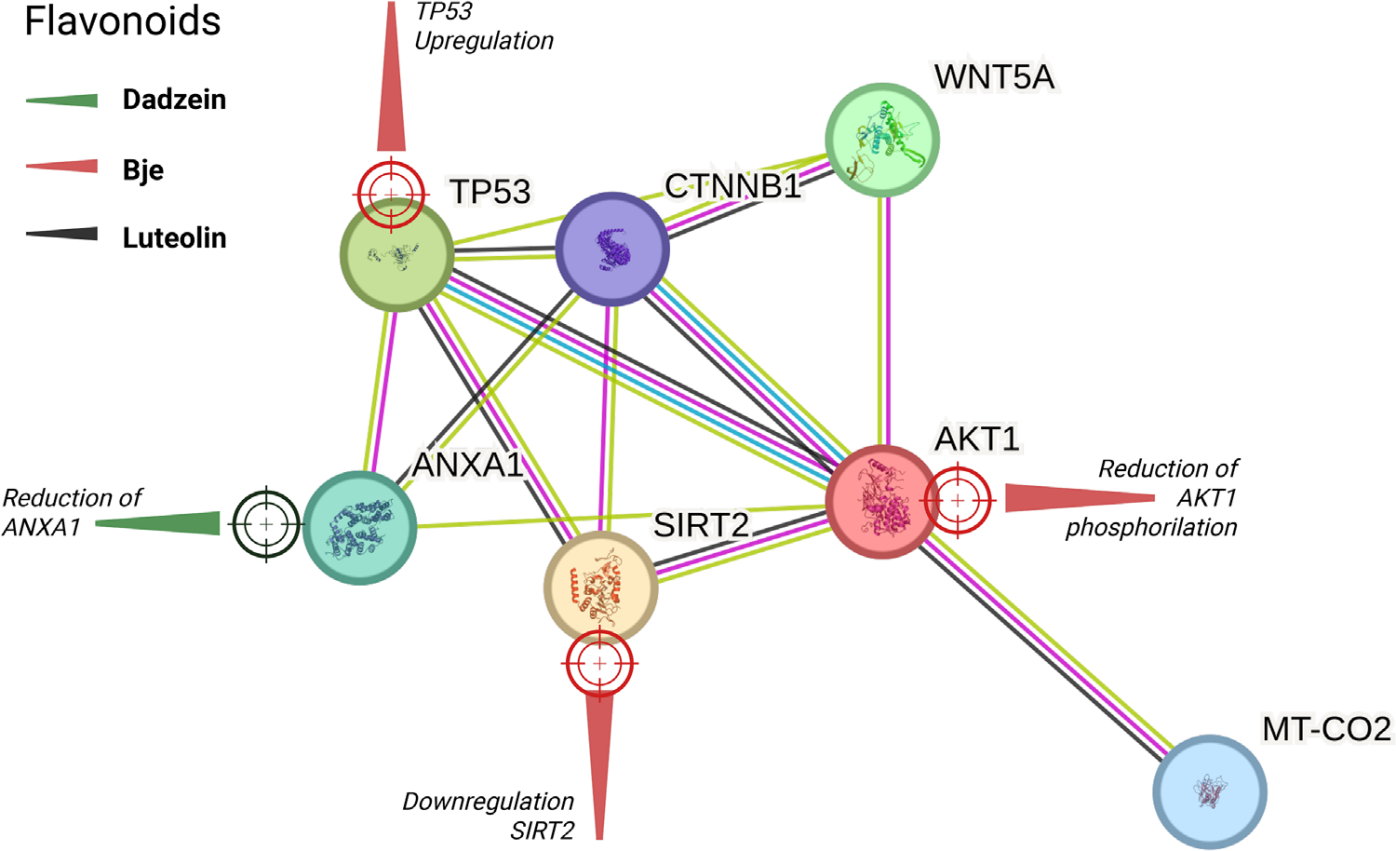
STRING [40] platform in silico data of interactions among flavonoid targets. An integrated network involving AKT1, TP53, SIRT2, WNT5A, and β‐catenin (CTNNB1) is observed, highlighting central axes related to cell signaling, survival, metabolism, and stress response.

Computational (in silico) data support virtual interactions among the flavonoid targets (Table S1), indicating a relationship among AKT1, TP53, SIRT2, and WNT5A [40] (Figure 2; Table S2). In a central hub, β‐catenin (CTNNB1) can be observed acting as a major interaction nexus, connecting to all six other proteins. TP53–AKT interaction (0.994). Another very strong piece of evidence links the tumor suppressor TP53 to the pro‐survival AKT kinase. WNT signaling is directly linked to β‐catenin and AKT, key components of the WNT signaling pathway. Finally, the SIRT2–TP53–AKT axis indicates a central role for SIRT2 (NAD‐dependent deacetylase) that bridges TP53 and AKT, suggesting roles in cell cycle, metabolism, and stress responses.

The overall analysis of epigenetic pathways on the other hand, as summarized in Table S3, supports a model in which flavonoids connect signal transduction pathway alterations (P19–TP53–PMicro21, caspase cascade, ERK/JNK, JAK2/STAT5, and autophagy machinery) to epigenetic mechanisms (DNMT downregulation, HDAC inhibition, histone acetylation increases, *SHP‐1* demethylation, and lncRNA H19 downregulation), culminating in cell‐cycle arrest, apoptosis, and/or autophagy‐linked cytoprotective effects [33, 41, 42, 43, 44].

The Flavonoids have been consistently linked to epigenetic regulation via DNMT enzymes [33, 41, 44]. EGCG [41] and baicalein [44] have been associated with DNMT1 downregulation. EGCG has a proven impact on G1 cell‐cycle arrest and on increased apoptosis through TP53 modulation. Baicalein, with additional *SHP‐1* demethylation, modulates JAK2/STAT5 signaling and induces apoptosis [44].

In parallel, quercetin is linked to downregulation of DNMT1 and DNMT3a and to increased H3 and H4 acetylation, a pattern consistent with transcriptionally permissive chromatin states in the ERK and JNK pathways, with increased histone acetylation as part of the associated remodeling. These integrated signaling and epigenetic shifts correspond to apoptosis induction [33].

Another epigenetic mechanism linked to flavonoids is the downregulation of exosomal lncRNA H19 induced by pure total flavonoids from citrus (PTFC). The PTFC is associated with protective effects on the intestinal mucosal barrier while also acting as an autophagy inducer, increasing the expression of light chain 3 (LC3) components (LC3‐I, LC3‐II) and tight‐junction markers (ZO‐1, occludin, claudin‐1) [43].

Overall, the evidence indicates that pathway‐level modulation by flavonoids leads to clear cellular effects, including reduced cell viability, with key pathways such as PI3K/Akt frequently implicated. In this context, differences in molecular effects among subclasses become particularly apparent when analyzing IFs.

### 1.3 IFs as Epigenetic Modulators in Different Pathways

**FIGURE 3:**
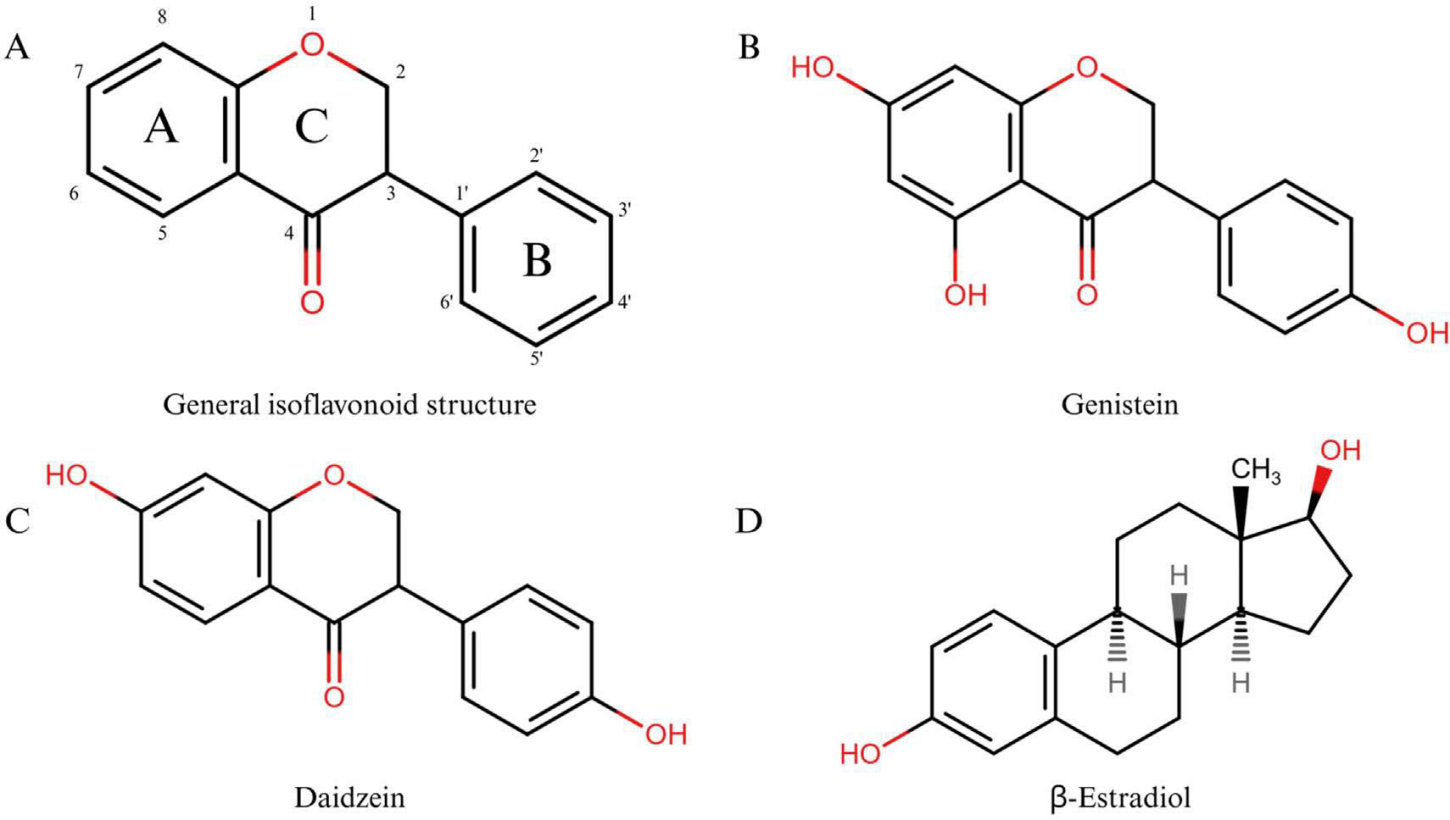
Chemical structure similarity between the general isoflavonoid structure (A), genistein (B), and daidzein (C), and mammalian endogenous hormone β‐estradiol (D). The only chemical structural difference between isoflavonoids and other flavonoids is their B ring at the C3 position of the heterocyclic C ring of the diphenylpropane structure (C6–C3–C6).

IFs are a subgroup of flavonoids predominantly found in soybeans and other legumes that exhibit great therapeutic potential against numerous diseases [22, 45]. This subclass comprises about 1000 known structures and is found among species of the subfamily Papilionoideae DC, which is considered a chemotaxonomic feature for this group [34]. Most IFs have structural similarities to endogenous mammalian β‐estradiol (estrogen) [22, 46] (Figure 3). Due to this similarity, IFs can selectively bind to estrogen receptors (ERs), conferring estrogen‐like activity that contributes to anticancer activity in hormone‐dependent cancers.

The IFs have meaningful anticancer and cytoprotective effects through epigenetic modulation, as reported in Table S3. The epigenetic pathways described for IF are different from those reported for flavonoids. MicroRNAs (miRNAs) are mainly described as involved in epigenetic mechanisms modulated by IF. However, DNA methylation pathways remain an important pathway described in the IF.

Genistein stands out within the IF class for its epigenetic regulatory activity. It has been associated with upregulation of miR‐23b, leading to inhibition of cell growth [47], and with downregulation of DNMT3B via genistein–miR‐29b–loaded hybrid nanoparticles, targeting the PI3K/AKT pathway and inducing antiproliferative effects, including apoptosis [48]. Genistein has also been linked to modulation of the Wnt signaling pathway, with molecular alterations including WNT5A upregulation and β‐catenin downregulation, alongside KMT5A upregulation and enrichment of the histone mark H4K20me1. Functionally, these changes were associated with G2/M cell‐cycle arrest, suggesting that genistein may remodel Wnt signaling and modulate chromatin‐associated regulation to block progression into mitosis [49].

Similarly, Irigenin regulates the miR‐425/RIPK1 pathway by upregulating miR‐425, effectively suppressing apoptosis, inflammation, and oxidative stress [50]. On the other hand, Puerarin modulates the TRPM3/miR‐204/Runx2 axis by downregulating TRPM3 and miR‐204 and activating Runx2, thereby enhancing cell proliferation, differentiation, and mineralization [51].

Micro RNAs (miRNAs) are small ncRNAs, approximately 22 nucleotides long, that play crucial tasks in gene regulation [52]. A variety of miRNAs could take specific signatures and important roles in the cancer progression, metastasis, diagnosis, and therapy in different cancer types such as colorectal [53, 54], pancreatic [55], breast [56], prostate [57], lung cancer [58], and leukemias [59, 60].

IFs have been used to treat and prevent several types of cancer due to their antiproliferative properties [61]. Over the past two decades, growing evidence has demonstrated that IFs modulate epigenetic mechanisms, thereby altering gene expression [62, 63]. These compounds influence epigenetic regulation through multiple interconnected mechanisms.

One of the key mechanisms involves the promotion of receptor dimerization and subsequent recruitment of nuclear co‐activator (NCoA) or co‐repressor (NCoR) complexes associated with histone HATs or HDACs. In addition, IFs modulate transcriptional activity by regulating DNA methylation patterns, which control the accessibility of the transcriptional machinery to specific genomic loci. They also participate in the activation and repression of histone marks by dynamically influencing chromatin‐modifying complexes at gene promoter regions. Furthermore, IFs affect the expression of tumor suppressor or oncogenic miRNAs and have been shown to downregulate the long noncoding RNA (lncRNA) HOTAIR, which is frequently overexpressed in cancer cells through miRNA‐mediated pathways [62, 64]. Collectively, these mechanisms highlight the multifaceted epigenetic potential of IFs in modulating gene expression programs involved in cancer development and progression.

The involvement of miRNAs in IF‐mediated regulatory processes has been examined in greater depth, given the importance of this compound class in epigenetic regulatory mechanisms [47, 50, 51], which seem particularly associated with other IF groups, such as Pterocarpans.

### 1.4 Pterocarpans

**FIGURE 4:**
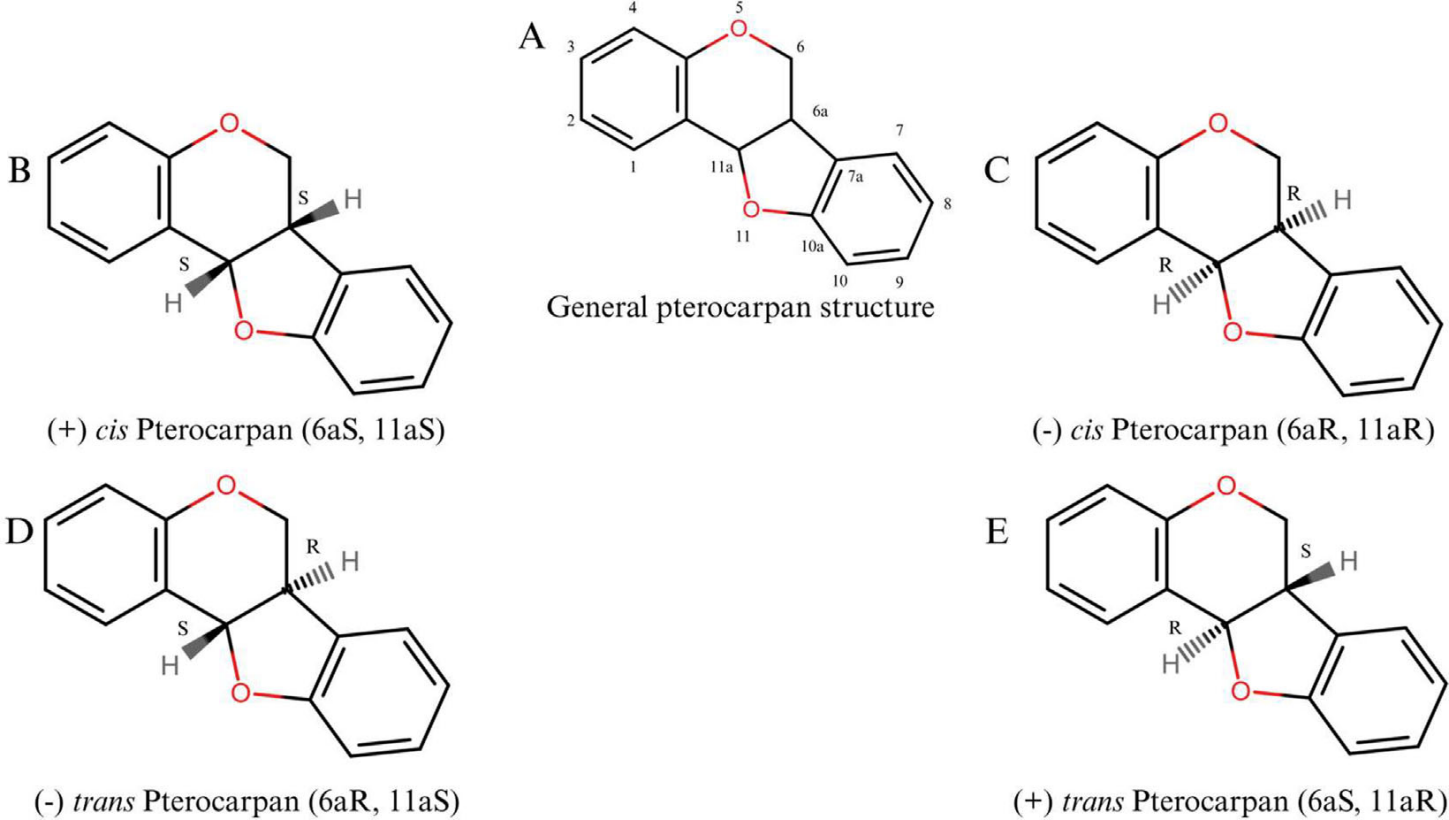
General structure of pterocarpans and their stereochemical possibilities.

Pterocarpans constitute the second‐largest group of IFs. The structure presents a tetracyclic nucleus derived from the basic IFs scaffold and is characterized by a fused heterocyclic benzo‐pyran‐furan‐benzene system that can be formed from isoflavones [65, 66]. The general structure of a pterocarpan and its possible stereochemical forms are presented in Figure 4. These compounds have a tetracyclic benzofuran‐benzopyran ring system with two chiral centers at positions 6a and 11a, which determines the stereochemical configuration of the molecule, although it is known that only naturally occurring compounds exhibit *cis*‐fusion of the B/C rings [66].

Pterocarpans are frequently used in traditional medicine as alternative and complementary therapies and have been extensively studied due to their numerous biological properties. A pterocarpan derivative isolated from the roots of *Sophora flavescens* Ait has been reported to have anti‐inflammatory properties [67].

A pterocarpan derived from *Erythrina subumbrans* (Hassk.) Merr. showed promising antidiabetic activity by inhibiting α‐amylase, as well as a potent antimicrobial activity against Gram‐positive bacterial strains, including *Enterococcus faecalis* ATCC 29212, *Micrococcus luteus* DMST 15503, methicillin‐resistant *Staphylococcus aureus* NPRC 001R, *S. aureus* ATCC 25923, and *Streptococcus pyogenes* ATCC 19615) and antifungal activity against *Candida albicans* ATCC 10231 [68].

Several other studies highlight that different pterocarpan molecules have demonstrated cytotoxic potential in various cancers, such as leukemia (HL‐60, Molt‐4, Jurkat, K562), breast (MCF‐7, BT‐549), colon (HCT‐116), melanoma (B16), prostate (DU 145, PC3), and ovarian cancer (OVCAR‐8, A2780) [61, 62, 63, 64, 65, 66, 67, 68, 69, 70, 71, 72].

Like other flavonoids, pterocarpans have also been reported as epigenetic agents, with different signaling pathways involved. PTFC modulate epigenetic effects by negatively regulating the expression of the lncRNA RNA H19 in exosomes derived from intestinal tissue. Regulation of the PI3K/AKT/mTOR signaling pathway induces autophagy and influences gene expression related to Atg5 and cellular cross‐talk proteins, thereby enhancing intestinal barrier protection against NSAID‐induced injury [43]. Irigenin upregulates the expression of miR‐425, which negatively regulates the expression of the receptor‐interacting protein kinase 1 (*RIPK1*) gene. This epigenetic modification leads to cardioprotection and cellular outcomes that favor cardiomyocyte survival and function [50].

Puerarin stimulates epigenetic effects by downregulating the TRPM3/miR‐204 signaling pathway, which contributes to increased expression of *RUNX2*, an *MC3T3‐E1* osteoblast protective factor, thus affecting osteogenic cell fate [51]. Genistein, an IF, exerts epigenetic effects via the PI3K/AKT signaling pathway by downregulating oncoproteins such as pAKT, p‐PI3K, DNMT3B, and MCL1. Genistein promotes apoptosis and inhibits proliferation in A549 lung cancer cells [48]. In MCF‐7 breast cancer cells, genistein significantly upregulates the expression of miR‐23b, leading to oncogenic gene repression, apoptosis induction, and antitumor response support [47].

Medicarpin, a natural pterocarpan, negatively regulates miR‐542‐3p, which inhibits bone morphogenetic protein 7 (BMP‐7) and PI3K/survivin signaling. This suggests that miR‐542‐3p suppresses osteogenic differentiation and promotes osteoblast apoptosis by repressing BMP‐7 and its downstream signaling [73]. In addition, medicarpin upregulates lipolytic gene expression of hormone‐sensitive lipase (HSL) and adipose triglyceride lipase (*ATGL*) by activating the cAMP/PKA/HSL signaling pathway in brown adipocytes, thereby stimulating protein phosphorylation and promoting lipolysis [74].

**FIGURE 5:**
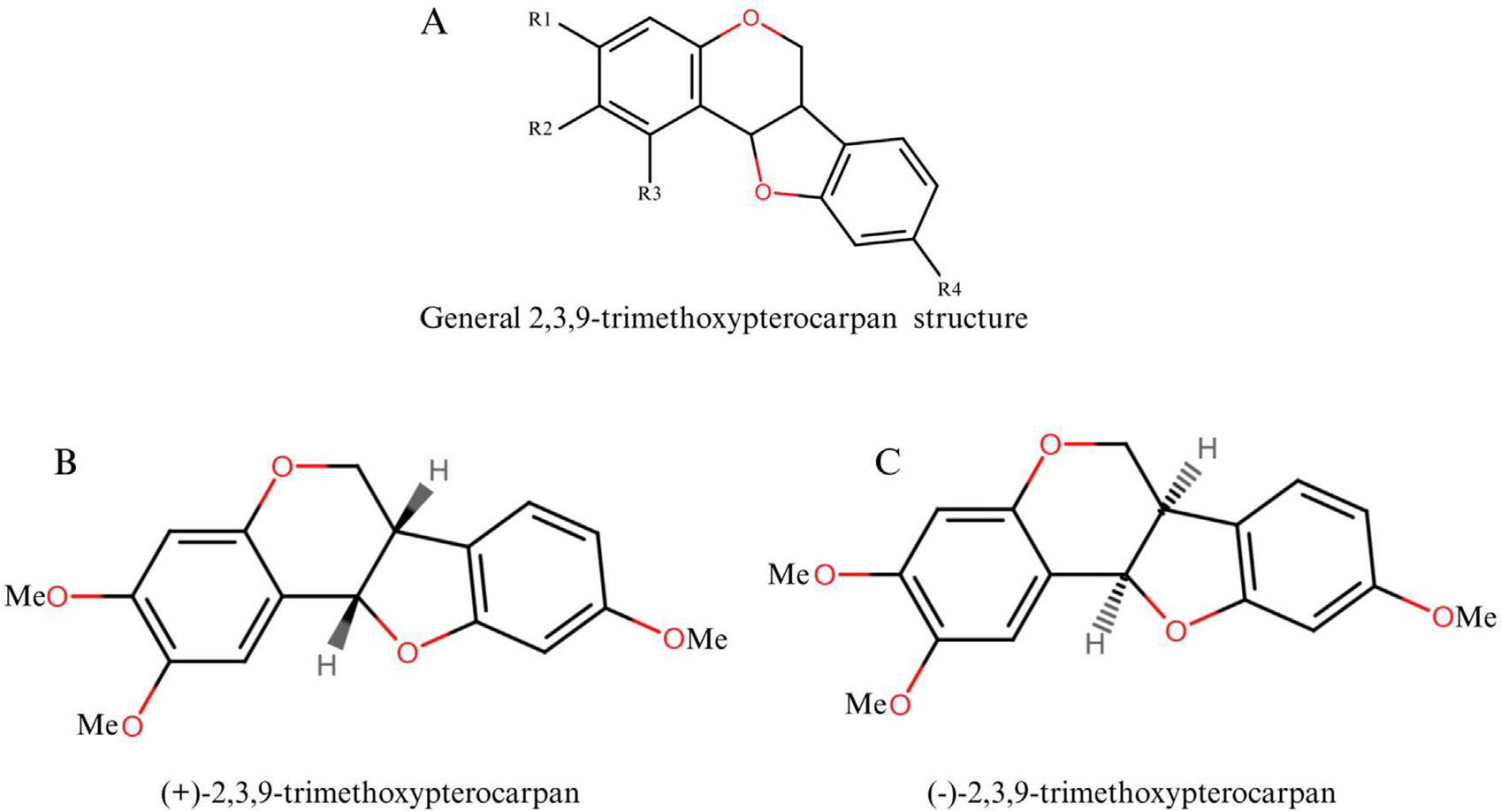
General formula of 2,3,9‐trimethoxypterocarpan (A): R1, R2, R3, and R4 are independently chosen from the group comprising OH; CH_3_O; and enantiomers (+) (B) and (−) (C).

Over the past two decades, our Brazilian research group has focused on the identification and characterization of pterocarpans (Figure 5A), especially (+)2,3,9‐trimetoxipterocarpan ((+)‐PTC) (Figure 5B,C), a natural enantiomer isolated from *Platymiscium floribundum* Vogel [75] that was first synthesized in 2012. The (+)‐PTC molecule has shown cytotoxic activity against four leukemia cell lines (HL‐60, MOLM‐4, JURKAT, and K562) and also appeared to be selective for tumor cell lines after showing a small reduction in the number of viable peripheral blood mononuclear cells (PBMC) (19% after 72 h) [76].

(+)‐PTC acts as an antimitotic agent by blocking the separation of duplicated centrosomes, a critical process in the formation of normal mitotic spindles, leading to cell cycle arrest during the transition to G2/M phase in leukemic cells. This cytostatic activity of (+)‐PTC in leukemic cells was confirmed by immunocytochemical experiments on the MCF‐7, T47D, and HS58T (human breast adenocarcinoma) cell lines [61].

All of these findings support the use of (+)‐PTC as a valid drug option based on an epigenetic approach. Table S3 summarizes the association between flavonoid derivatives and epigenetic modifications. All the studied structures are described in terms of their chemical aspects in Table S4.

### 1.5 Common Molecular Pathways Associated With Flavonoids, IFs, and Pterocarpans

In addition to their epigenetic role, flavonoids, IFs, and pterocarpans also regulate other common molecular pathways (Table S1). This is also important, as different signaling mechanisms contribute to the development of distinct types of cancer. Several studies have reported multiple effects, including morphological differentiation, cell cycle arrest, cytotoxicity with apoptosis/necrosis induction, cell proliferation, and reduced cell viability.

The relevant participation of TP53 in the pathways regulated by flavonoids, IFs, and pterocarpans highlights an upstream signaling control that converges into coordinated cell cycle block and programmed cell death (Table S1). The flavonoids have been described to induce mitochondrial apoptosis (with an increase in BAX and a reduction in BCL2), ANXA1 reduction, and SIRT2/AKT/TP53 signaling, which align with cell‐cycle arrest and apoptosis/DNA fragmentation outcomes [32, 35, 36].

The IFs shown emphasize growth/survival circuitry (VEGF/CXCL12/CXCR4) and also highlight the PI3K/AKT pathway leading to reduced viability or decreased proliferation [46, 77]. Pterocarpans share the same phenotype of cytotoxicity/apoptosis, but map stress and checkpoint networks. Sarno et al. [78] observed that medicarpin extracted from *Trifolium repens* L. causes inhibition of cell growth associated with complete inhibition of BCR‐ABL/STAT5 and activation of the P^38^ signaling pathway. Hancio et al. [79]. described the LQB‑118 compound's potential to induce cell death in cytarabine‑resistant cells by reducing the expression of the XIAP protein. In addition, it was also observed that *AURKB* downregulation led to morphological changes typical of the cell death process, as well as the cell cycle arrest in the G2/M phase and apoptosis induction [80].

### 1.6 In Silico ADME Prediction to Evaluated Compounds

Given the molecular modulation relevance, the pharmacological potential of the molecules previously described in this study (flavonoids, IFs, and pterocarpans) was assessed using their predicted ADME values to determine pharmacokinetic properties (Table S5). These properties were calculated using SwissADME [81] (Swiss Institute of Bioinformatics). The designs of the molecules were created using the MolView application [82] (Bergwerf Labs) (see Supporting Information Material S1 for chemical structures and SMILES results). The potential of a compound as a drug is strongly influenced by its ADME properties [83].

The use of software capable of predicting ADME of new drug candidate molecules is a practical advantage in early drug development, as it enables in silico evaluation without the need for experimental data [84]. ADME predictions aid in the rational selection of viable candidates prior to synthesis by providing rapid, cost‐effective, and reliable insights [85].

**FIGURE 6:**
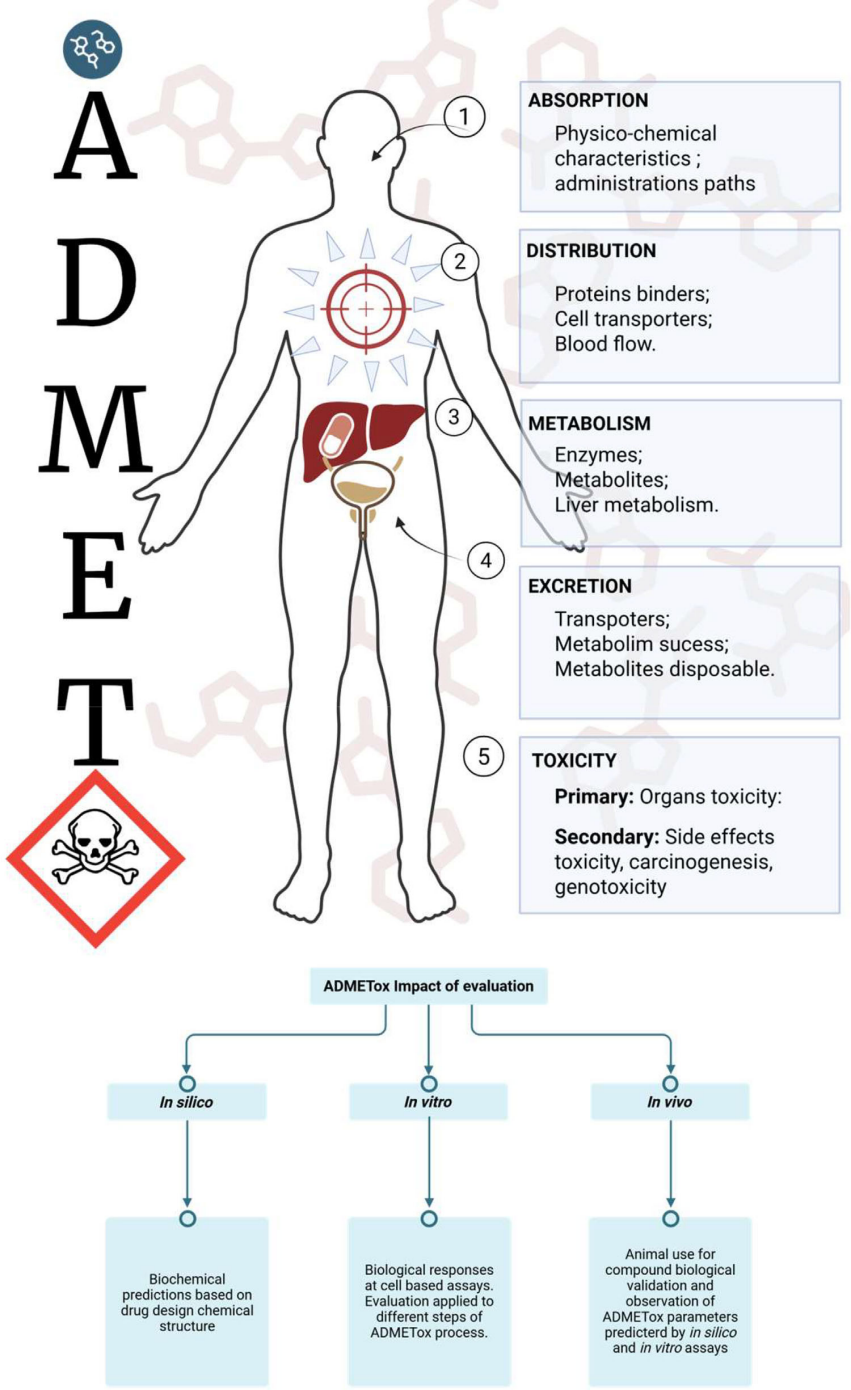
Schematic representation of ADMET processes and their evaluation and its components: absorption (physicochemical properties and administration routes), distribution (protein binding, cell transporters, blood flow), metabolism (enzymes, metabolites, liver metabolism), excretion (transporters, metabolic success, metabolite disposal), and toxicity (primary organ toxicity and secondary adverse effects such as side effects, carcinogenesis, and genotoxicity). The lower panel summarizes the approaches used for ADMET assessment: in silico (computational predictions based on drug structure), in vitro (biological responses evaluated in isolated cells or tissues), and in vivo (animal models for systemic and toxicological evaluation).

In silico ADME predictions evaluate multiple parameters to assess a molecule's performance (Figure 6). Pharmacokinetics refers to the processes of absorption, distribution, metabolism, and excretion without toxic effects. A promising candidate must have a predictable metabolism that does not produce toxic metabolites. Physicochemical properties such as molecular weight, number of heavy atoms, and polarity influence the ability of a molecule to cross biological membranes and be orally absorbed [81], which is crucial for AML therapies targeting the bone marrow and hematopoietic system. Good solubility, usually expressed as the logarithm of molar solubility (log *S*), is essential for gastrointestinal absorption and systemic bioavailability and can be predicted by different models (ESOL, Ali, Silicos‐IT). Drug‐likeness parameters, including PAINS alerts and Lipinski violations, are based on efficacy/safety comparisons with thousands of known drugs [81, 86]. A candidate molecule that violates many of these parameters must be avoided. In addition, promising molecules must be chemically stable and commercially viable (i.e., synthetically accessible).

**FIGURE 7:**
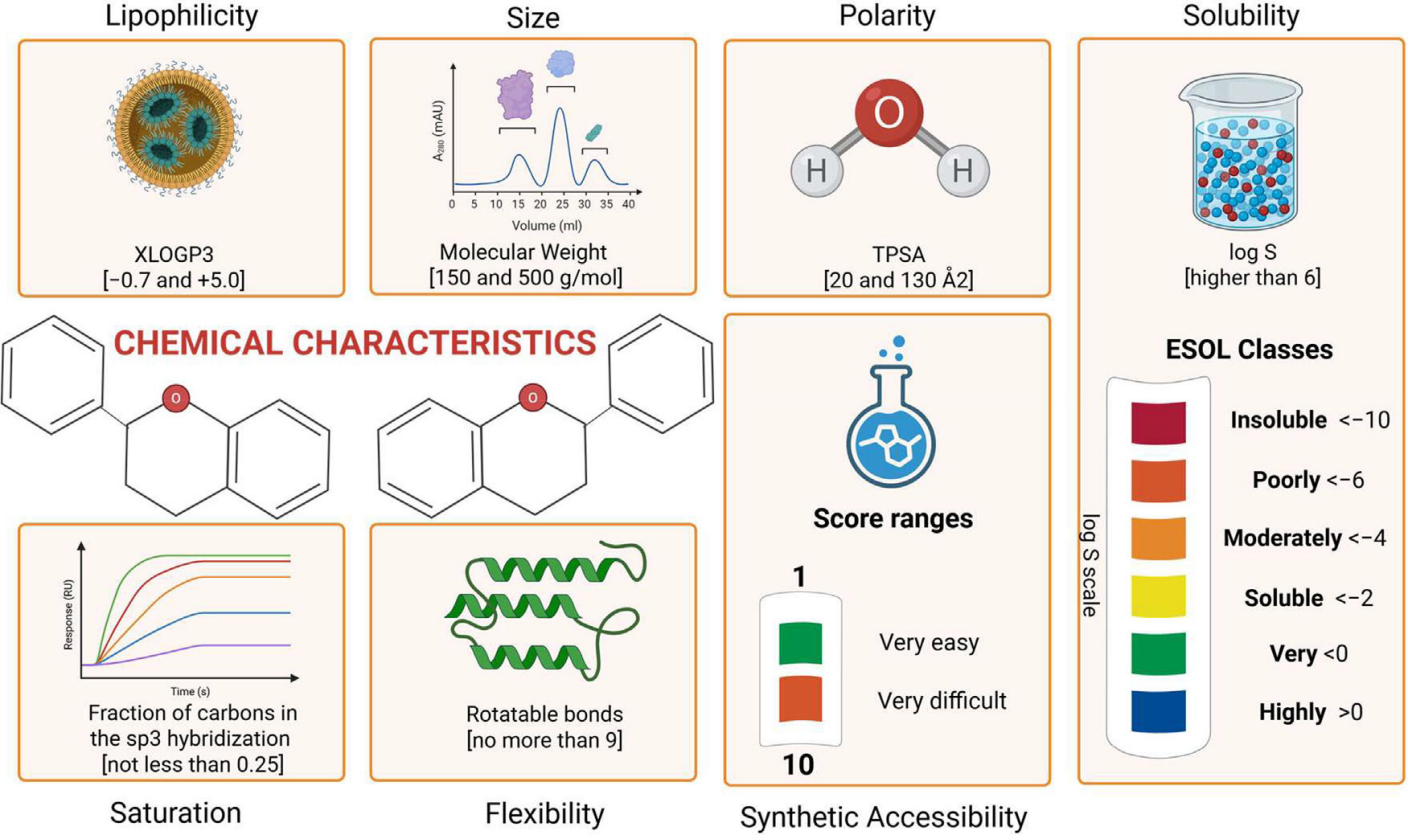
Bioavailability radar criteria and cutoffs for screening candidate compounds. Key chemical characteristics influencing drug‐likeness and pharmacokinetic behavior. Parameters include lipophilicity (XLOGP3), molecular size (molecular weight between 150 and 500 g/mol, ≤ 9 hydrogen bond donors/acceptors), polarity (TPSA 20–130 Å^2^), and aqueous solubility (log *S* > −6, classified by ESOL classes). Additional features comprise saturation (fraction of carbons in sp^3^ hybridization ≥ 0.25), molecular flexibility (number of rotatable bonds ≤ 9), and synthetic accessibility, scored from 1 (*very easy*) to 10 (*very difficult*).

The bioavailability radar criteria [81] were considered most relevant for screening candidate compounds for AML treatment (see Supporting Information Material S2 for radar evaluation results). In this context, Daina et al. [81] and Martin [87] established the following criteria shown in Figure 7: (a) lipophilicity refers to the affinity of the compound for nonpolar environments. Values in the optimal range indicate favorable oral absorption and systemic bioavailability. (b) Size refers to the molecular weight of a compound (g/mol), corresponding to a range that supports membrane permeability and oral activity, defined by the bioavailability radar and the Lipinski's rule of five; (c) polarity refers to the surface area occupied by polar atoms (oxygen and nitrogen). The values are related to the permeability of the gastrointestinal tract and the blood–brain barrier (BBB); (d) solubility refers to the solubility of a compound in water, a critical parameter for orally administered drugs; (e) saturation refers to the amount of sp^3^‐hybridized carbon atoms in the molecule; higher saturation values may reduce nonspecific interactions; (f) flexibility refers to the number of single bonds that confer conformational adaptability to the molecule, an important parameter for absorption and ligand binding specificity; (g) molecular weight, in accordance with the Lipinski rule, when below 500 g/mol, favors both permeability and solubility; (h) ESOL classes (log *S* scale) categorize aqueous solubility, which is useful for preliminary screening based on less complex formulation and more likely absorption; (i) synthetic accessibility (SA score) estimates the difficulty of synthesizing a molecule, taking into account structural complexity; and finally (j) a bioavailability score (ABS) is a measure to estimate the higher probability of oral bioavailability of molecules in vivo. ABS ranges from 0.11 (*very low probability*), 0.17 (*low probability*), 0.55 (*moderate probability*) if the molecule fulfills the Lipinski rule of five, 0.56 (*moderate probability*) to 0.85 (*high probability of oral bioavailability*).

In Table S5, medicarpin, LQB‐118, and (+)‐2,3,9‐trimethoxypterocarpan stand out for their high gastrointestinal absorption and ability to cross the BBB without violating Lipinski's rule, which is desirable in drug candidates for the treatment of leukemia. (+)‐2,3,9‐trimethoxypterocarpan, in particular, has good solubility and molecular weight, similar to the other molecules, and accessibility for synthesis within expectations. In fact, the first record of the synthesis of this molecule dates back more than a decade.

In Figure S1, the molecule (+)‐2,3,9‐trimethoxypterocarpan is identified as number 17. Its graph overlaps with the pink area, suggesting a strong likelihood of drug‐like properties. The BOILED‐Egg model in SwissADME supports the prediction for good intestinal absorption and BBB crossing. The blue dot indicates a probable substrate for P‐glycoprotein, which is related to its potential for active efflux from both the BBB and GI. These parameters altogether suggest that (+)‐2,3,9‐trimethoxypterocarpan is promising as an epigenetic drug.

In a biotechnological approach, optimizing ADME properties, as with many natural products, it is desirable to enhance the efficacy of flavonoids. In this context, accumulating evidence indicates that nanocarrier‐based strategies can enhance the therapeutic performance of flavonoids. An example is Isorhamnetin nanoformulations that have demonstrated increased cellular uptake and cancer cell targeting, thereby improving therapeutic efficacy through enhanced delivery mechanisms and modulation of critical cancer‐related molecular pathways [88].

## 2 Conclusions

This review highlights the potential of IFs to modulate epigenetic mechanisms that regulate gene expression. These small molecules may influence multiple molecular targets through key epigenetic processes, including the modulation of miRNA networks (miR‐23b, miR‐29b, miR‐425, and miR‐204) and DNA methylation (DNMT3B). In parallel, IFs have also been associated with chromatin‐related regulation, including modulation of chromatin marks (KMT5A) and enrichment of H4K20me1, with effects on pathways such as p19Arf–TP53–p21Cip1, PI3K/AKT/mTOR, TRPM3/miR‐204, cAMP/PKA/HSL, ERK, and JNK signaling, and caspase cascade activation.

Taken together, these findings suggest that IFs can reprogram cancer‐relevant phenotypes via miRNA signatures and epigenetic markers linked to DNA methylation and histone modifications. Given their favorable pharmacological characteristics, IFs emerge as a promising epigenetic‐based strategy that may contribute to the development of new therapeutic approaches against cancer, with particularly strong evidence in leukemia models.

## Conflicts of Interest

The authors declare no conflicts of interest.

## Supporting information




**Supporting File 1**: cbdv70979‐sup‐0001‐SuppMat.docx

**Supporting File 2**: cbdv70979‐sup‐0002‐FIgureS1.pdf

**Supporting File 2**: cbdv70979‐sup‐0003‐Tablw S1‐S5.xlsx

## Data Availability

The authors have nothing to report.
