# Supplementary material for: Isoflavonoids and Epigenetic Modulation: Therapeutic Insights for Cancer Treatment
Source: Chem Biodivers. 2026 Feb 16;23(2):e03446. doi: 10.1002/cbdv.202503446 (PMC12908931; doi:10.1002/cbdv.202503446)
Supplement: Supplementary file 1 — Supporting File 1: cbdv70979‐sup‐0001‐SuppMat.docx [file CBDV-23-e03446-s001.docx]

**SUPPLEMENTARY MATERIAL**

**SM Table 1** Assessment of flavonoid derivatives and their association to molecular mechanisms (*in vitro* studies) in biological systems.

| **Chemical class** | | **Pathway alteration** | **Molecular alteration** | **Cytological alteration** | **Reference** |
| --- | --- | --- | --- | --- | --- |
| Flavonoids | Luteolin | Apoptosis induction by the mitochondrial pathway | Bax upregulation, Bcl2 downregulation | Induction of morphological differentiation into granulocytes and DNA fragmentation | Ganai et al. (2021)^[32]^ |
|  | Daidzein | Reduction of ANXA1 levels | Downregulation of COX-2 activity | Cell cycle arrest, apoptosis, and phagocytosis | Sabran et al. (2021)^[35]^ |
|  | Flavonoid-Rich Extract of Bergamot Juice (BJe) | SIRT2/AKT/TP53 pathway | AKT phosphorylation reduction, SIRT2 downregulation, TP53 upregulation | Cell cycle arrest in S phase and apoptosis induction | Maugeri et al. (2022)^[36]^ |
| Isoflavonoids | Genistein | Wnt Signaling Pathway | Wnt5a upregulation, β-catenina downregulation, KMT5A upregulation, H4K20me1 enrichment rather than DNA demethylation | Cell cycle arrest in G2/M phase | Zhou et al. (2021)^[49]^ |
|  | Genistein | VEGF/CXCL12/CXCR4 | CXCR4 downregulation and enhancement of VEGF expression and secretion | Decrease cell viability in a time- and dose-dependent manner | Shahmoradi et al. (2022)^[77]^ |
| Pterocarpans | Medicarpin | BCR-ABL/STAT5 pathway | BCR/Abl downregulation | Strong cytotoxic effect with apoptosis/ necrosis induction | Sarno et al (2020)^[78]^ |
|  | LQB-118 | NFκB pathway | XIAP downregulation | Apoptosis induction | Hancio et al. (2021)^[79]^ |
|  | (+)-2, 3, 9-Trimethoxypterocarpan | CEP55, AURKB, MAD2, CDC20, ATM pathways | Downregulation of AURKB expression | Morphological alterations, cell cycle arrest, and apoptosis induction | Sales (2022)^[80]^ |

**SM Table 2** Assessment of flavonoid derivatives and their association to epigenetic mechanisms (*in vitro* studies) in biological systems.

| **Chemical class** | | **Pathway alteration** | **Epigenetic alteration** | **Cytological alteration** | **Reference** |
| --- | --- | --- | --- | --- | --- |
| Flavonoids | Epigallocatechin gallate (EGCG) | p19^Arf^-TP53-p21^Cip1^ signaling pathway | n, DNMT1 downregulation, and p19^Arf^, TP53 and p21^Cip1^ upregulation | Cell cycle arrest in G1 phase, increase in apoptosis | Wu et al. (2020)^[41]^ |
|  | Apigenin–Vorinostat-Conjugate (AVC) | Caspase cascade activation | HDACs 1, 2, 3, and 6 inhibition, p21^Cip1^ up-regulation, increase in H3 acetylation | Apoptosis at the early and late phases | Zhang et al. (2021)^[42]^ |
|  | Pure total flavonoids from Citrus (PTFC) | Evaluation of exosomes and autophagosomes pathways by the expression of light chain 3 (LC3)-I, LC3-II, ZO-1, Occludin and Claudin-1 | Downregulating exosomal lncRNA H19 | Protective effect on the intestinal mucosal barrier and an autophagy inducer. | Chen et al. (2024)^[43]^ |
|  | Quercetin | ERK and JNK pathways activation, increase in histone acetylation | DNMT1 and DMNT3a downregulation, increase in H3 and H4 acetylation | Apoptosis induction | Zhu et al. (2023)^[33]^ |
|  | Baicalein | JAK2/STAT5 signaling pathway | DNMT1 downregulation, SHP-1 demethylation | Apoptosis induction | Xu et al. (2023)^[44]^ |
| Isoflavonoids | Genistein | miR-23b regulation | Up-regulation of miR-23b | Inhibits cell growth | Avci et al. (2015)^[47]^ |
|  | Puerarin | TRPM3/miR-204/Runx2 | Downregulation of TRPM3/miR-204 and activation of Runx2. | Cell proliferation, differentiation and mineralization, | Zeng et al. (2018)^[51]^ |
|  | Genistein-miRNA-29b-loaded hybrid nanoparticles (GMLHN) | AKT/PI3K/DNMP3B | Downregulation of DNMT3B | Antiproliferative effect and apoptosis initiation (pAKT and p-PI3K pathway) | Sacko et al (2019)^[48]^ |
|  | Irigenin | miR-425 regulation/RIPK1 | Up-regulation of miR-425 | Suppress apoptosis, inflammation and oxidative stress | Guo et al. (2020)^[50]^ |
| Pterocarpans | Medicarpin | Protein Kinase A (PKA) | Interference RNA | Regulate the levels of hormone-sensitive lipase (Hsl) and adipose triglyceride lipase (Atgl) | Imran et al. (2018)^[74]^ |
|  | Medicarpin | BMP-7/PI3K- survivin signaling | miR-542-3p | Tumor repressor and inhibition of osteogenesis by BMP-7-mediated. | Kureel et al. (2014)^[73]^ |

**SM Table 3** STRING^[40]^ (STRING Consortium) *in silico* interaction data among flavonoid targets. Interaction scores, evidence types, and confidence levels highlight strong associations among AKT1, TP53, SIRT2, WNT5A, and β-catenin (CTNNB1) within key signaling pathways.

| **Protein A** | **Protein B** | **Score** | **Evidence type** | **Confidence of evidence** |
| --- | --- | --- | --- | --- |
| **TP53** | **AKT1** | 0.994 | Database, Text-mining, Experimental | Higest |
| **AKT1** | **CTNNB1** (β-catenin) | 0.979 | Database, Experimental, Text-mining | Higest |
| **TP53** | **CTNNB1** (β-catenin) | 0.927 | Text-mining | Higest |
| **SIRT2** | **CTNNB1** (β-catenin) | 0.900 | Experimental, Text-mining | Higest |
| **WNT5A** | **CTNNB1** (β-catenin) | 0.869 | Experimental, Text-mining | High |
| **SIRT2** | **AKT1** | 0.773 | Experimental, Text-mining | High |
| **SIRT2** | **TP53** | 0.768 | Text-mining | High |
| **TP53** | **ANXA1** | 0.762 | Experimental, Text-mining | High |
| **WNT5A** | **AKT1** | 0.750 | Experimental, Text-mining | High |
| **ANXA1** | **CTNNB1** (β-catenin) | 0.624 | Text-mining | Medium |
| **WNT5A** | **TP53** | 0.477 | Text-mining | Medium |
| **MT-CO2** | **AKT1** | 0.467 | Experimental, Text-mining | Medium |
| **ANXA1** | **AKT1** | 0.424 | Text-mining | Medium |
|  |  |  |  |  |

**SM Table 4** Molecule class, 2D structures and SwissADME canonical SMILES.

| **Class** | **Molecule** | **2D structure** | **Canonical SMILES** |  |
| --- | --- | --- | --- | --- |
| Flavonoids | General flavonoid structure | 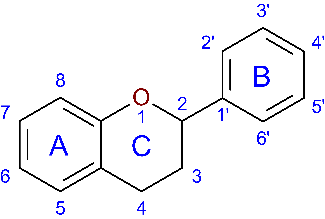 | c1cccc2CCC(Oc12)c1ccccc1 |  |
|  | Luteolin structure | 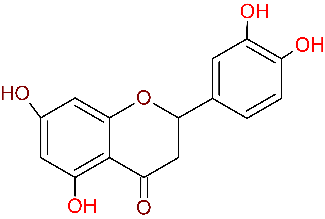 | O=C1CC(Oc2cc(O)cc(O)c21)c1cc(O)c(O)cc1 |  |
|  | Quercetin structure | 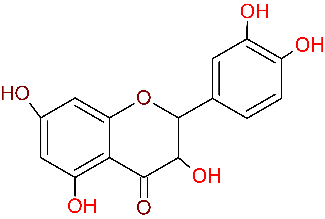 | OC1C(Oc2cc(O)cc(O)c2C1=O)c1cc(O)c(O)cc1 |  |
| Isoflavonoids | General isoflavonoid structure | 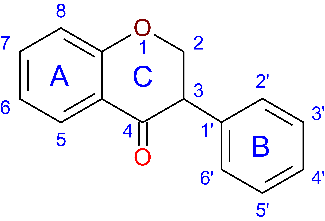 | O=C1C(COc2ccccc12)c1ccccc1 |  |
|  | Genistein structure | 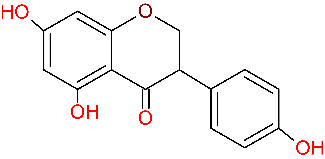 | O=C1c2c(cc(O)cc2O)OCC1c1ccc(O)cc1 |  |
|  | Daidzein structure | 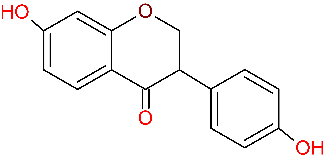 | O=C1C(COc2cc(O)ccc12)c1ccc(O)cc1 |  |
| Pterocarpans | General pterocarpan structure | 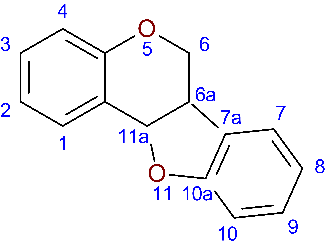 | C1Oc2ccccc2[C@H]2Oc3ccccc3[C@@H]12 |  |
|  | (+) cis pterocarpan 6aS 11aS structure | 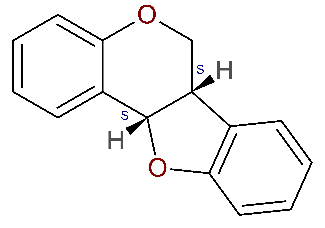 | C1Oc2ccccc2[C@H]2Oc3ccccc3[C@@H]12 |  |
|  | (-) cis pterocarpan 6aR 11aR structure | 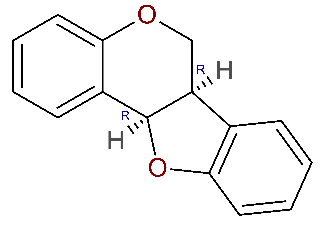 | C1Oc2ccccc2[C@@H]2Oc3ccccc3[C@H]12 |  |
|  | (-) trans pterocarpan 6aR 11aS structure | 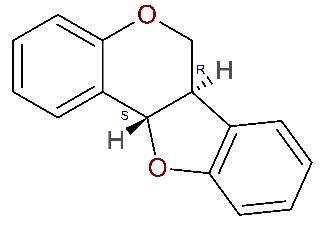 | C1Oc2ccccc2[C@H]2Oc3ccccc3[C@H]12 |  |
|  | (+) trans pterocarpan 6aS 11aR structure | 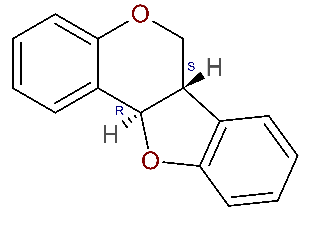 | C1Oc2ccccc2[C@@H]2Oc3ccccc3[C@@H]12 |  |
|  | Medicarpin structure | 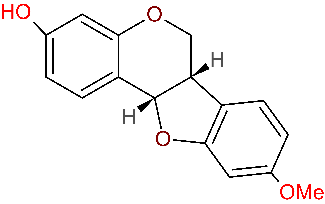 | Oc1cc2OC[C@H]3[C@H](Oc4cc(ccc43)OC)c2cc1 |  |
|  | LQB-118 structure | 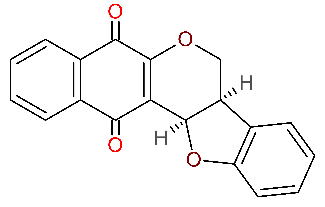 | O=C1C=2OC[C@@H]3[C@@H](Oc4ccccc43)C=2C(=O)c2ccccc12 |  |
|  | General 2,3,9-trimethoxypterocarpan structure | 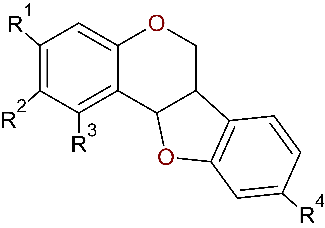 | [*]c1cc2OC3c4c([*])c([*])c([*])cc4OCC3c2cc1 |  |
|  | (+)2,3,9-trimethoxypterocarpan structure | 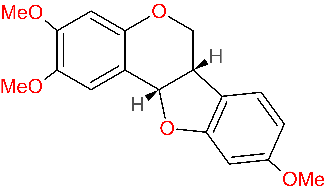 | COc1cc2OC[C@H]3[C@H](Oc4cc(ccc43)OC)c2cc1OC |  |
|  | (-)2,3,9-trimethoxypterocarpan structure | 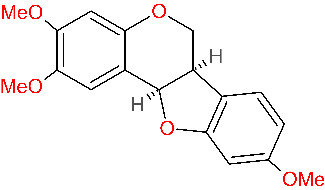 | COc1cc2OC[C@@H]3[C@@H](Oc4cc(ccc43)OC)c2cc1OC |  |

**SM Table 5** ADME prediction of different flavonoid, isoflavonoid, and pterocarpan molecules. *according to Lipinski et al.^[86]^ and Daina et al.^[81]^.

|  | **Physicochemical Properties** | | **Water Solubility** | | **Pharmacokinetics** | | **Druglikeness** | | **Medicinal Chemistry** | | |
| --- | --- | --- | --- | --- | --- | --- | --- | --- | --- | --- | --- |
| **Molecule name** | **Formula** | **Molecular Weight (g/mol)** | **ESOL Log S (mol/L)** | **ESOL Class** | **GI absorption** | **BBB permeant** | **Lipinski violations** | **ABS** | **Pains alerts (Ideal = 0*)** | **Bren alerts (Ideal = 0*)** | **Synthetic Accessibility** |
|  |  |  |  |  |  |  | **(Ideal = 0*)** |  |  |  |  |
| Luteolin | C15H10O6 | 286.24 | -3.71 | Soluble | High | No | 0.00 | 0.55 | 1.00 | 1.00 | 3.02 |
| Quercetin | C15H10O7 | 302.24 | -3.16 | Soluble | High | No | 0.00 | 0.55 | 1.00 | 1.00 | 3.23 |
| Genistein | C15H10O5 | 270.24 | -3.72 | Soluble | High | No | 0.00 | 0.55 | 0.00 | 0.00 | 2.87 |
| Daidzein | C15H10O4 | 254.24 | -3.53 | Soluble | High | Yes | 0.00 | 0.55 | 0.00 | 0.00 | 2.79 |
| Medicarpin | C15H12O4 | 256.25 | -3.43 | Soluble | High | Yes | 0.00 | 0.55 | 0.00 | 0.00 | 3.43 |
| LQB-118 | C19H12O4 | 304.30 | -3.84 | Soluble | High | Yes | 0.00 | 0.85 | 1.00 | 0.00 | 4.12 |
| (+)-2, 3, 9-Trimethoxypterocarpan | C18H18O5 | 314.33 | -3.91 | Soluble | High | Yes | 0.00 | 0.55 | 0.00 | 0.00 | 3.88 |


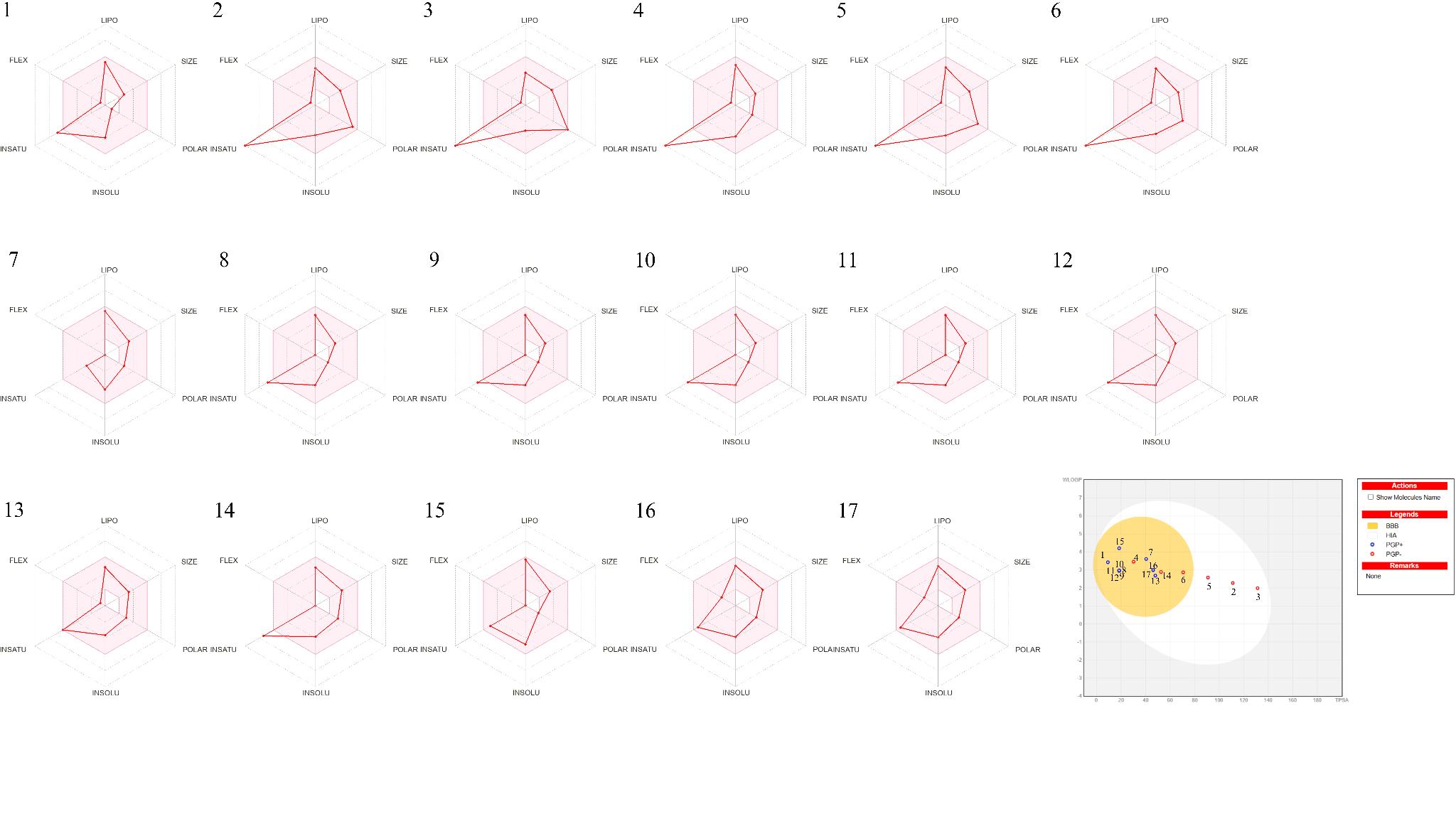
**SM Figure 1** ADME BoiledEGG and radar for all the flavonoids, isoflavonoids and pterocarpans presented in the review.
