# Supplementary figures and images for: Isoflavonoids and Epigenetic Modulation: Therapeutic Insights for Cancer Treatment
Source: Chem Biodivers. 2026 Feb 16;23(2):e03446. doi: 10.1002/cbdv.202503446 (PMC12908931; doi:10.1002/cbdv.202503446)

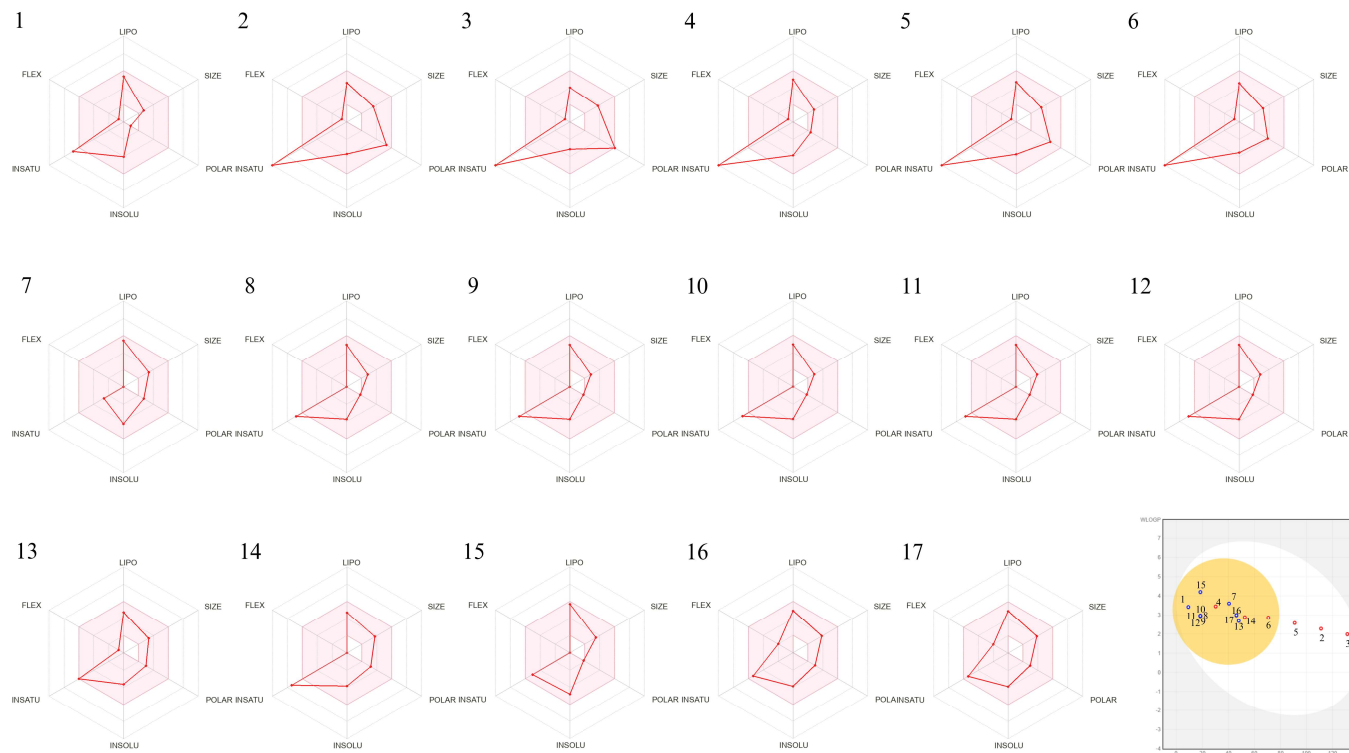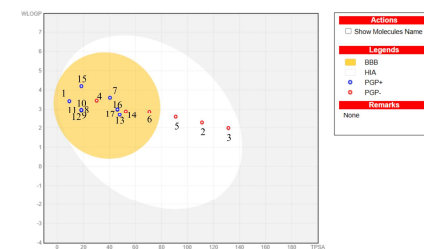

Supplement: Supplementary file 2 — Supporting File 2: cbdv70979‐sup‐0002‐FIgureS1.pdf [file CBDV-23-e03446-s003.pdf]
